# Genome-Wide Association Study of Immune Indices in Yaks

**DOI:** 10.3390/ani15142114

**Published:** 2025-07-17

**Authors:** Daoning Yu, Xiaoming Ma, Chun Huang, Tong Wang, Mengfan Zhang, Fen Feng, Xiaoyun Wu, Yongfu La, Xian Guo, Ping Yan, Derong Zhang, Chunnian Liang

**Affiliations:** 1 Key Laboratory of Animal Genetics and Breeding on Tibetan Plateau, Ministry of Agriculture and Rural Affairs, Lanzhou 730050, China; ydn9907@163.com (D.Y.); maxiaoming@caas.cn (X.M.); johnchun825@163.com (C.H.); wgeniust@163.com (T.W.); zmf13664139695@163.com (M.Z.); feng990111@163.com (F.F.); wuxiaoyun@caas.cn (X.W.); layongfu@caas.cn (Y.L.); guoxian@caas.cn (X.G.); pingyanlz@163.com (P.Y.); 2 Key Laboratory for Yak Genetics, Breeding, and Reproduction Engineering of Gansu Province, Chinese Academy of Agricultural Sciences, Lanzhou 730050, China; 3 College of Life Sciences and Engineering, Northwest Minzu University, Lanzhou 730106, China

**Keywords:** yak, immune indices, resequencing, GWAS, SNP

## Abstract

The yak is a vital livestock resource on the Qinghai–Tibet Plateau, renowned for its strong disease resistance and high-quality meat. However, various diseases pose significant threats to its health and lead to substantial economic losses. Current feeding management practices, along with available drugs and vaccines, have demonstrated limited effectiveness in preventing and controlling infectious diseases. Additionally, challenges such as drug resistance and the safety of animal products persist. Therefore, enhancing the disease-resistant breeding capacity of yaks is crucial. In this study, we examined 192 yaks by measuring the concentrations of 10 immune indicators in serum by using the ELISA method and conducting whole-genome resequencing, which identified 19,182,942 SNP loci. Through genome-wide association analysis, we detected 323 significant SNPs located near or within 125 candidate genes, most of which are associated with disease and significantly enriched in the TGF-β signaling pathway. Overall, our study identified a series of novel variants and candidate genes associated with disease resistance traits in yaks, providing important information for the molecular breeding of disease resistance in yaks. These results not only contribute to a deeper understanding of the function of disease resistance genes in yaks but also hold great potential for accelerating precision disease resistance breeding in yaks.

## Simple Summary

Yaks are important animals for people living on the Qinghai–Tibet Plateau because they provide meat and other resources. However, sick yaks can cause huge losses for farmers. The current ways to prevent these diseases, such as medicines and vaccines, do not always work well and can have problems like drug resistance and safety concerns. In this study, we observed 192 yaks and measured substances in their blood that show how their immune system works. We also studied the yaks’ DNA to find small genetic differences linked to disease resistance. We found many new genetic markers and genes that help yaks fight disease, especially those involved in a key biological pathway related to immune responses. These discoveries can help us breed yaks that are naturally better at resisting diseases, which will improve the health of yak populations and benefit farmers by reducing losses and the need for medicines.

## 1. Introduction

Immune indicators are important criteria for assessing an animal’s disease resistance, primarily reflecting the health status and function of the immune system. The core responsibility of the immune system is to identify and eliminate pathogens that invade the body, such as bacteria and viruses. When pathogens invade, the immune system quickly initiates a series of immune responses to resist and eliminate these foreign threats. Immune indicators encompass various parameters, such as antibody levels, immune cell counts, inflammation markers, and cytokines. Antibodies are produced by B cells and are specific, effectively binding to and neutralizing pathogens, thereby enhancing the overall efficiency of the immune response [1]. Additionally, the quantity and function of immune cells are important indicators for assessing the health status of the immune system. When faced with pathogen invasion, immune cells are rapidly activated and begin to proliferate to enhance the response to infection [2]. At the same time, the levels of inflammation markers and cytokines also reflect the state of the immune system’s response, helping to monitor and regulate the intensity and duration of the immune response, ensuring that the immune system can effectively cope with challenges and maintain overall health [3,4]. Disease resistance is influenced by a combination of factors, including genetics [5,6], environment [7], nutrition [8,9], and age [10]. For example, genetic factors may determine the structure and function of an animal’s immune system, while environmental conditions and nutritional status can significantly affect the health and responsiveness of the immune system.

Consequently, immune indices can serve as significant references for assessing an animal’s disease resistance capacity and health status. Through monitoring variations in immune indices, it is feasible to promptly identify an animal’s health condition and adopt corresponding measures to enhance its disease resistance ability, effectively preventing and treating diseases. Screening for variation in loci related to disease resistance at the genetic level and conducting association analyses to reveal the relationships between genes and traits enable the utilization of genetics and genomics for early selection. This will conspicuously improve the efficiency and accuracy of selection, presenting broad application prospects. Existing studies have reported associations between some animal genes and immune indices. In livestock research, Chen et al. [11] identified multiple genes associated with natural antibodies and discovered that the level of natural antibodies (NAb) in the blood of healthy piglets is hereditary and potentially serves as a latent genetic indicator for resisting various microbial diseases. Lin et al. [12] identified 14 candidate genes related to the concentration of colostrum and serum immunoglobulin in dairy cows via genome-wide association analysis (GWAS), laying the groundwork for elucidating the key genes and causal mutations influencing the immunoglobulin concentration in colostrum and providing crucial information for the genetic improvement of immune-related traits in dairy cows. In poultry, studies have revealed that 39 SNPs are associated with 6 immune traits, such as total serum IgY, and are linked to 5 genes, which might play a pivotal role in regulating immune responses. The discovery of these candidate genes offers important clues for the further identification of immune-related genes and provides potent information for uncovering the molecular mechanisms of disease resistance traits in chickens [13].

Yaks (*Bos grunniens*), native to the Qinghai–Tibet Plateau and its surrounding regions in China, thrive in extreme environments characterized by high altitudes, cold temperatures, significant diurnal temperature fluctuations, a short pasture growth period, intense ultraviolet radiation, and low oxygen levels. These unique ecological conditions have endowed yaks with remarkable adaptability, as evidenced by their cold resilience, heat tolerance, delayed maturity, low reproductive rates, and ability to survive in high-altitude, hypoxic conditions [14,15,16,17]. However, diseases pose a significant challenge to yak health and productivity within the yak farming industry, resulting in substantial economic losses [18,19]. Although the incidence of disease can be somewhat mitigated through improved breeding management and the use of pharmaceuticals and vaccines, achieving complete control over infectious diseases remains a formidable challenge. Furthermore, the widespread use of pharmaceuticals raises concerns regarding drug resistance, which could compromise the safety of livestock products [20,21]. In the long term, understanding the genetic basis of disease resistance and identifying genes associated with disease resilience could facilitate molecular-level breeding for enhanced disease resistance. Such advances are expected to improve yak immunity and resilience to disease, thereby addressing health challenges in a sustainable way. Disease-resistant breeding not only has significant potential for economic benefits, but the resistant animals it produces can also provide important animal models for human disease research, promoting scientific progress in this field [22,23]. With the development of molecular biology, genetics, and genetic engineering, disease resistance breeding is playing a more and more important role in yak breeding. Therefore, investigating the genes associated with yak immune markers is essential for advancing disease-resistance breeding and enhancing disease prevention and control. In this study, we conducted a genome-wide association analysis on the serum concentrations of 10 immune markers in the Niangya yak population in Tibet, aiming to identify functional genes, elucidate their associations with immune traits, and provide a molecular foundation for enhancing disease resistance in yaks through selective breeding.

## 2. Materials and Methods

### 2.1. Sample Collection

In November 2023, blood samples were collected from 192 healthy adult female yaks raised under standardized farming conditions at the Niangya Yak Farm in Jiali County, Naqu City, Tibet Autonomous Region, with no clinical signs of significant diseases observed. Whole blood (5 mL) was collected via venipuncture and placed in a clean pro-coagulation vacuum collection vessel for 30 min, then centrifuged at 3500 r/min for 10 min. The supernatant was sucked into a PE tube, and then sealed and stored in a low-temperature freezer at −20 °C for the detection of immune indexes. Another 5 mL blood sample was collected and frozen in the freezer at −20 °C for subsequent DNA extraction.

### 2.2. Determination of Immune Indicators

The IgA (MB-4907A), IgG (MB-4616A), IgM (MB-4908A), CRP (MB-2161B), HP (MB-2158B), IL2 (MB-4923B), IL4 (MB-4904B), IL6 (MB-4905B), IFN-γ (MB-4902B), and TNF-α (MB-4838B) assay kits from Jiangsu Meibiao Biotechnology Co., Ltd., Nanjing, China, were used for measurements, following the double-antibody, one-step sandwich method. After equilibration at room temperature for 20 min, the required strips were removed from the aluminum foil pouch, and the remaining strips were resealed in a zip-lock bag and stored at 4 °C. Standard wells and sample wells were set up, with 50 μL of standards at various concentrations to each standard well. For the sample wells, 10 μL of test sample was added, followed by 40 μL of sample diluent, while the blank wells were left empty. Then, 100 μL of horseradish peroxidase (HRP)-conjugated detection antibody was added to each standard and sample well. The plate was covered with a sealer and incubated at 37 °C for 60 min. After incubation, the liquid was discarded and the plate was blotted dry with absorbent paper. Each well was completely filled with Wash Solution, allowed to stand for 1 min, and then emptied and blotted dry. This washing step was repeated five times. Next, 50 μL of both Chromogen Solution A and Chromogen Solution B was added to each well, gently mixed, and incubated at 37 °C for 15 min in the dark. Then 50 μL of Stop Solution was added to each well, causing the color to change from blue to yellow. If the color appeared green or uneven, the plate was gently tapped to ensure thorough mixing. The Optical Density (O.D.) of each well was measured at 450 nm using a microplate reader within 15 min. Finally, a standard curve was constructed by plotting the standard concentrations on the *x*-axis and the corresponding O.D. values on the *y*-axis in Excel. A linear regression curve was generated, and its equation was used to calculate the concentration of immune markers in each sample.

### 2.3. Genotyping and Quality Control

Whole-genome resequencing was conducted using a TIANamp Blood DNA Kit (Tian Gen Biotech Co. Ltd., Beijing, China) to extract DNA. The DNA concentration was measured using a Qubit Fluorometer (Thermo Fisher Scientific, Waltham, MA, USA), and the integrity of DNA samples was assessed via 1% agarose gel electrophoresis. Samples that passed the test were broken using ultrasound and fragmented with magnetic beads to concentrate the sample fragments to a size range of 300–400 bp. Sequencing libraries were prepared and sequenced on the DNBSEQ platform (BGI-Shenzhen, Shenzhen, China) after PCR and purification. For data processing, the raw sequencing data were first quality controlled and filtered using SOAPnuke software (version 2.2.1) [24]. Subsequently, the mem algorithm of BWA software (version 0.7.17) [25] was employed to align the filtered high-quality reads to the reference genome (LU_Bosgru_v3.0). The alignment results were then converted into sorted BAM files by using Samtools software (version 1.9) [26]. Quality assessment and statistical analysis of the generated BAM files were performed by using the bamqc tool (version 2.2.2-dev) from Qualimap2 software [27]. For single-nucleotide polymorphism (SNP) detection, we utilized GATK software (version 4.2.6.1) [28] to identify SNP loci with default parameters, followed by the functional annotation of the SNPs by using SnpEff software (version: 5.2c) [29]. To further enhance data quality, we applied Plink software (version 1.90) [30] for the quality control of the SNP data, employing specific filtering criteria of –geno 0.1, –maf 0.01, and –hwe 1 × 10^−6^ to exclude low-quality SNPs.

### 2.4. Population Genetic Analysis

Principal component analysis (PCA) was conducted on the filtered SNP data by using Plink (version 1.90). The extracted significant feature vectors were utilized for mapping to observe individual clustering trends. To assess relatedness, we calculated the relatedness matrix among all samples by using the VanRaden algorithm implemented in GCTA (version 1.93) [31]. Linkage disequilibrium (LD) decay was analyzed using PopLDdecay (version 3.42) [32] to determine the LD coefficient (r^2^) for paired SNPs.

### 2.5. Genome-Wide Association Analysis (GWAS)

GWAS employs the Fixed and Random Model Circulating Probability Unification (FarmCPU) algorithm from rMVP [33] to identify SNPs. This algorithm iteratively utilizes the Fixed Effect Model (FEM) and the Random Effect Model (REM). Initially, FarmCPU conducts a screening of all markers by using the Fixed Effect Model, incorporating potential association loci as covariates in the model. Subsequently, these potential association loci are optimally predicted by a Random Effect Model, which is continuously updated in each iteration. This process ensures the simultaneous control of false positives and enhances detection accuracy by alternating between Fixed and Random Effect Models, ultimately improving the identification of genetic associations for complex traits. The Fixed Effect Model was as follows:

$$y_{i}=M_{i1}b_{1}+M_{i2}b_{2}+\cdots +M_{it}b_{t}+S_{ij}d_{j}+e_{i}$$

In this model, $y_{i}$ represents the observed value for the i-th individual; M denotes the genotypes of t possible associated loci included in the model, which is initially empty at the start of the iterative process; b signifies the effect value of the loci with potential associations incorporated into the model; $S_{ij}$ indicates the genotype of the j-th genetic marker for the i-th individual; and $d_{j}$ represents the effect value of the genotype at the corresponding locus of $S_{ij}$. $e_{i}$ is the residual vector. The Random Effect Model is formulated as follows:

$$y_{i}=u_{i}+e_{i}$$

where $u_{i}$ is the total genetic effect of the i-th individual [34].

SNPs exhibiting a −log10(*p*-value) greater than 6 (i.e., *p* < 1 × 10^−6^) after establishing a fixed threshold were considered to have statistically significant associations. Although a Bonferroni correction for the 19,182,942 variants would yield a threshold of approximately 2.61 × 10^−9^ (0.05/19,182,942), we adopted the threshold of *p* < 1 × 10^−6^ based on prior studies that demonstrated its effectiveness in balancing type I error control with the risk of false negatives [35]. Population stratification can lead to spurious association results in genome-wide association studies (GWASs). Therefore, we calculated the genomic inflation factor (λ) and plotted quantile–quantile (Q-Q) plots to assess the stratification of our study population.

### 2.6. Gene Annotation and Enrichment Analysis

To identify candidate genes linked to target traits, significant SNPs were annotated using SnpEff (version: 5.2c) based on the reference genome (LUBosgru_v3.0, GCA_005887515.1). Prior to gene annotation, the physical distance corresponding to the decay of the average linkage disequilibrium coefficient to below 0.1 was calculated. Based on the results of LD analysis, we identified the genes nearest to the association loci as potential candidate genes. In addition, to further resolve the biological functions of these genes, gene ontology (GO) functional enrichment analysis was performed using the DAVID bioinformatics resource (https://david.ncifcrf.gov/, accessed on 5 December 2024) and Kyoto Gene and Genome Encyclopedia (KEGG) pathway enrichment analysis. In the enrichment analysis, *p* < 0.05 was used as the criterion for determining the significant enrichment of GO terms and KEGG pathways.

## 3. Results

### 3.1. Descriptive Statistics for Immune Indicators

**Figure 1:**
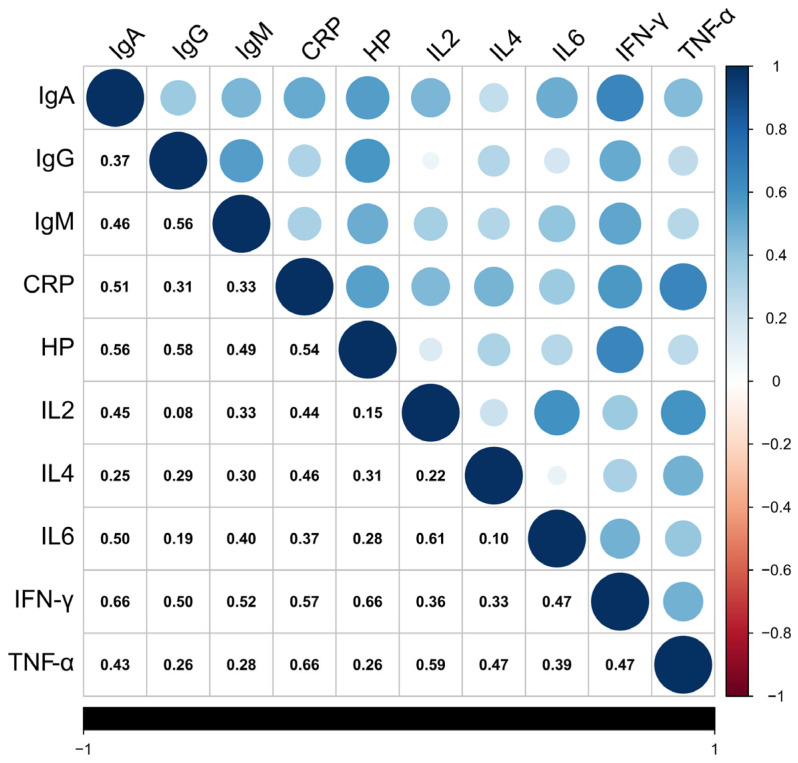
Heat map of correlation coefficient matrices for 10 immune traits.

We derived descriptive statistics and conducted phenotypic analyses of 10 traits, including humoral immunity (IgA, IgG, and IgM), inflammatory indicators (CRP, HP), and cytokines (IL2, IL4, IL6, IFN-γ, and TNF-α) in a sample of 192 yaks (Table 1 and Table S1). The coefficients of variation for each trait ranged from 12.17% to 22.35%, with IgG exhibiting the highest coefficient of variation and IgM the lowest. Further correlation analyses were performed on all individual immune traits, revealing significant positive correlations among most traits, with Pearson’s correlation coefficients ranging from 0.10 to 0.66 (Figure 1). The strongest correlations were observed between IFN-γ and HP, as well as between TNF-α and CRP. Additionally, IFN-γ demonstrated strong correlations with other traits.

### 3.2. Whole-Genome Sequencing Data

We conducted whole-genome resequencing on 192 yaks, and the raw reads generated were stored in the FASTQ file format for subsequent analysis. The average sequencing coverage depth across all samples was 11×, resulting in a total of 3.89 Tb of raw reads, with an average of 19.65 Gb per sample. The sequencing quality was high, with an average score of 98.64% for Q20 and 94.77% for Q30 following quality control. The GC content of the 192 samples ranged from 42.34% to 44.8%, indicating that the library construction and sequencing processes did not exhibit significant bias. After data quality control, we identified a total of 19,182,942 SNP loci. Their distribution across chromosomes is illustrated in Supplementary Figure S1, while the SNP annotation results are presented in Supplementary Table S2. The annotation analysis revealed that 51.13% of the SNPs were located in intronic regions, 38.58% in intergenic regions, 4.46% in upstream regions, 4.39% in downstream regions, and only 0.14% of the SNPs were found in exonic regions.

### 3.3. Population Genetic Diversity Analysis

**Figure 2:**
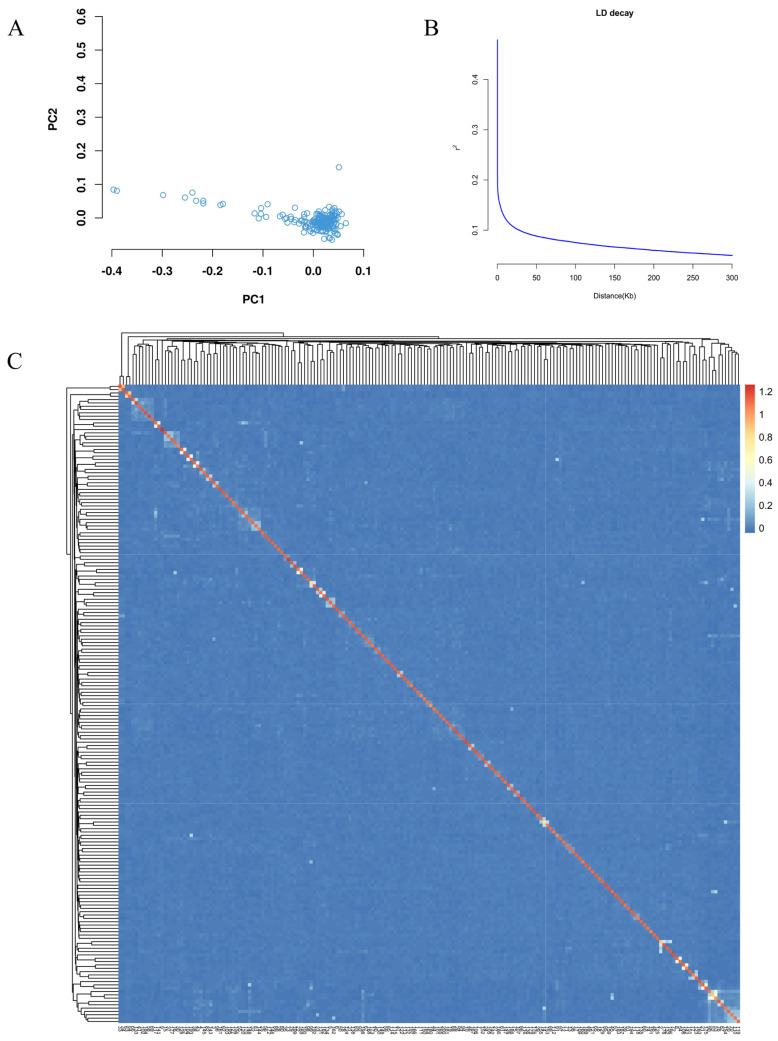
(**A**). Principal component analysis results. (**B**). Linkage disequilibrium (LD) decay analysis. (**C**) Results of G-matrix analysis of genetic relationships among Niangya yak populations. The horizontal and vertical coordinates are 192 individual Niangya yaks. Each small square represents the genetic relationship between two individuals. The closer the color is to red, the closer the genetic relationship between two individuals is, and vice versa.

We conducted principal component analysis (PCA) on 19,182,942 single-nucleotide polymorphisms (SNPs) to investigate the population structure of 192 yaks. The PCA results illustrated the relationships between the first two principal components, revealing that most individuals clustered closely together, with only a few exhibiting outlier distributions. This pattern suggests that the populations possess a relatively homogeneous genetic background (Figure 2A). After understanding the affinities of the population, we further constructed a genomic relationship matrix (G-matrix) based on genome-wide markers and performed genomic affinity analysis (Figure 2C). The results showed that most of the inter-individual kinships were in the lower to middle degree, indicating a low degree of inbreeding within the population. The linkage disequilibrium (LD) decay curve is shown in Figure 2B, which corresponds to a decay distance of approximately 30 kb when the LD coefficient decays to 0.1. Therefore, we searched for potential key genes within the region of 30 kb upstream and downstream of the significant locus, which may be associated with the target traits, and initially served as candidate genes for subsequent studies.

### 3.4. Genome-Wide Association Analysis of Immune Indicators

**Figure 3:**
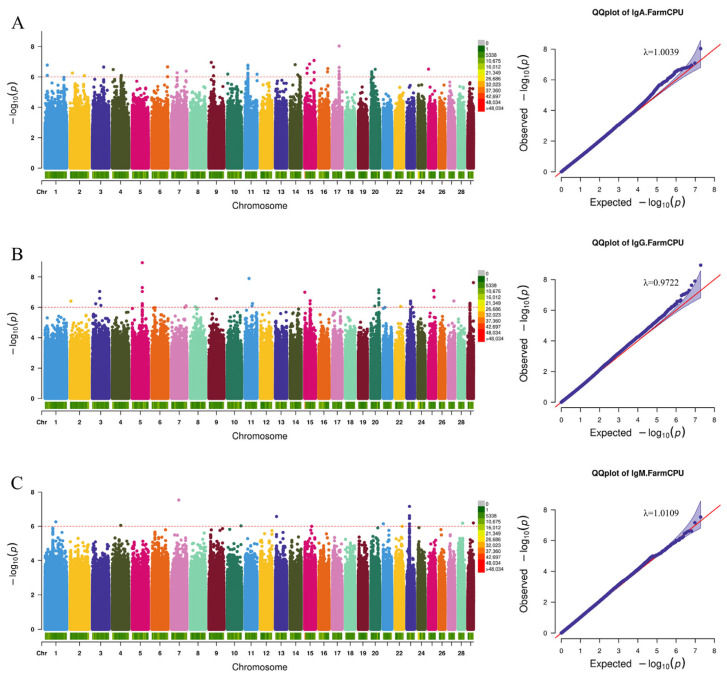
Manhattan and QQ plots of humoral immune correlations. (**A**) Immunoglobulin A, (**B**) Immunoglobulin G, and (**C**) Immunoglobulin M. The red dashed line is the threshold of −log10 (*p*-value), and points beyond the threshold range are significantly correlated sites.

**Figure 4:**
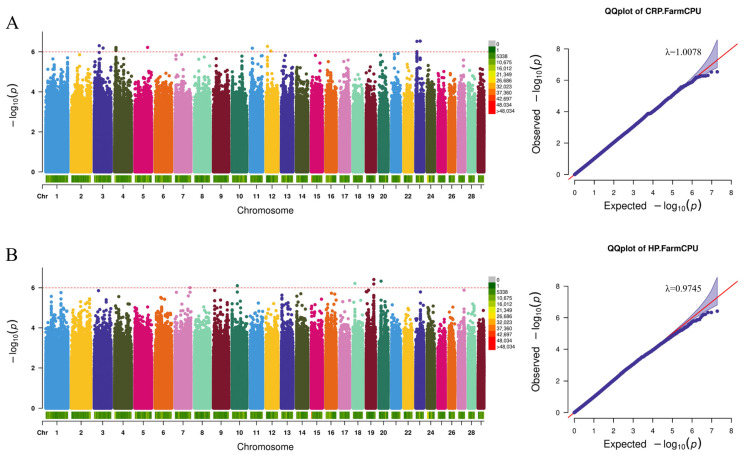
Manhattan and QQ plots related to inflammation indicators. (**A**) C-reactive protein, (**B**) Haptoglobin. The red dashed line is the threshold of −log10 (*p*-value), and points beyond the threshold range are significantly correlated sites.

**Figure 5:**
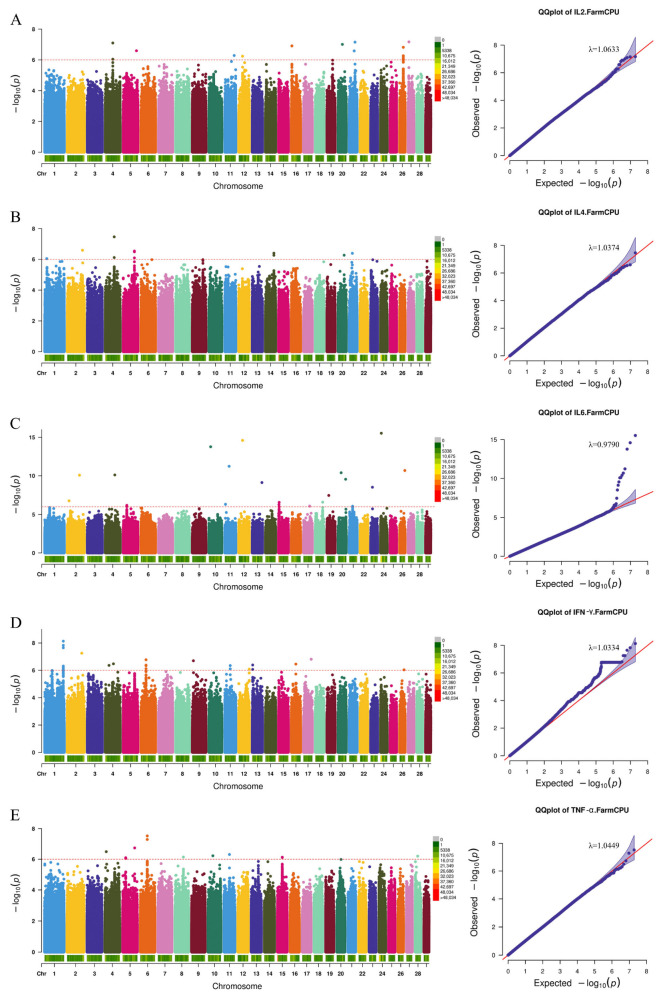
Cytokine-related Manhattan and QQ plots. (**A**) Interleukin-2, (**B**) Interleukin-4, (**C**) Interleukin-6, (**D**) Interferon γ, and (**E**) Tumor necrosis factor α. The red dashed line is the threshold of −log10 (*p*-value), and points beyond the threshold range are significantly correlated sites.

By analyzing the detected SNPs in relation to humoral immunity, inflammatory indicators, and cytokines, we successfully identified several genetic markers and candidate genes that are closely associated with the target traits. As illustrated in the QQ plots (Figure 3, Figure 4 and Figure 5) and the genomic inflation factors (λ = 0.97–1.06), no population stratification was observed.

In the context of humoral immunity-related indicators, we identified a total of 119 mutations in immunoglobulin-related SNPs. Among these, 54 SNPs were associated with Immunoglobulin A (IgA), 49 SNPs with Immunoglobulin G (IgG), and 16 SNPs with Immunoglobulin M (IgM) (Figure 3 and Supplementary Table S3). Through gene annotation, we identified 64 related genes. Notably, the SNP associated with IgA (Chr14:39923048 G>A) was located in the exonic region of the Castor zinc finger 1 (*CASZ1*) gene. Additionally, three SNPs associated with IgG were all found in the intronic region of the EGF containing fibulin extracellular matrix protein 1 (*EFEMP1*) gene. Variants of IgM-related SNPs, on the other hand, were annotated to six genes, including Lipoma-preferred partner (*LPP*), Integrin alpha9 (*ITGA9*), and Myotubularin-related protein 7 (*MTMR7*).

In the analysis of inflammation-related indicators, a total of 23 SNP mutations were identified and annotated to 15 genes. Among these, three significant SNPs associated with C-reactive protein (CRP) were found in Lysine Methyltransferase 2C (*KMT2C*), PTPRF Interacting Protein Alpha 2 (*PPFIA2*), and within the intronic region of Solute Carrier Family 6 Member 18 (*SLC6A18*). Additionally, SNPs associated with Haptoglobin (HP) were annotated to genes such as Failed Axon Connection Homolog (*FAXC*) and Ribosomal Protein S6 Kinase B1 (*RPS6KB1*) (Figure 4 and Supplementary Table S3).

In the cytokine association analysis, we identified 19 SNPs associated with Interleukin-2 (IL2), 11 SNPs associated with Interleukin-4 (IL4), 25 SNPs associated with Interleukin-6 (IL6), and an additional 19 SNPs associated with Interleukin-2 (IL2). A total of 113 SNPs were associated with Interferon γ (IFN-γ), of which 91 SNPs were annotated to the intron region of Adhesion G Protein-Coupled Receptor L3 (*ADGRL3*). Furthermore, we identified 13 SNPs associated with Tumor necrosis factor α (TNF-α). In total, 46 genes were annotated, including Deleted in Colorectal Cancer (*DCC*), Ceramide Kinase-Like Protein (*CERKL*), Thrombospondin Type 1 Domain Containing 4 (*THSD4*), and Bicaudal D homolog 1 (*BICD1*), among others (Figure 5 and Supplementary Table S3).

### 3.5. Functional Enrichment Analysis of Candidate Genes

**Figure 6:**
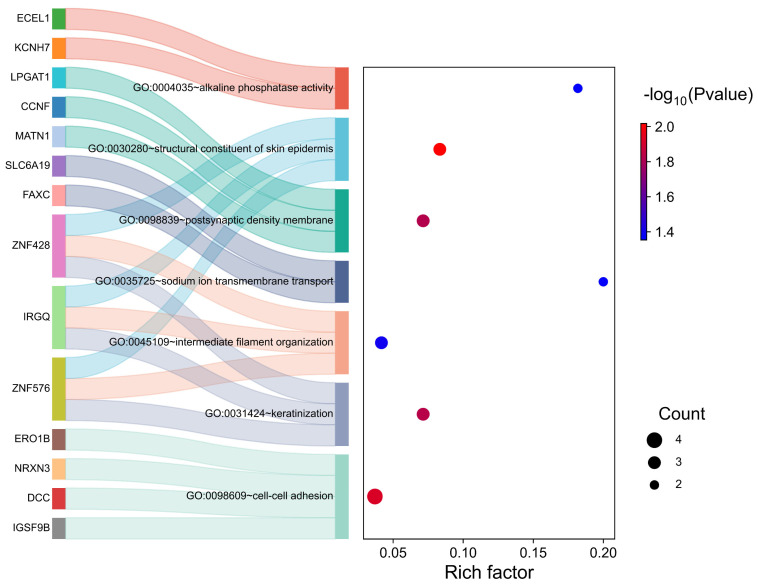
GO enrichment analysis of genes at significantly associated loci.

To further investigate the functional and regulatory relationships of these significant SNP markers and their respective candidate genes, we conducted a functional enrichment analysis of 125 genes by using the GO and KEGG databases. The GO analysis revealed seven significantly enriched functional terms (*p* < 0.05): cell–cell adhesion (GO:0098609), keratinization (GO:0031424), intermediate filament organization (GO:004510), sodium ion transmembrane transport (GO:0035725), postsynaptic density membrane (GO:0098839), structural constituent of skin epidermis (GO:0030280), and alkaline phosphatase activity (GO:0004035). The corresponding genes are presented in Figure 6 and Supplementary Table S4. In the KEGG enrichment analysis, only one significant pathway, the TGF-beta signaling pathway, was identified (Supplementary Table S5), with the enriched genes in this pathway including *THSD4* and *RPS6KB1*.

## 4. Discussion

As animals living at high altitude, yaks are resistant to stress and can adapt to extreme living conditions. However, the genetic mechanisms underlying the immune traits associated with stress resistance have rarely been investigated. Studying these mechanisms will not only enhance our understanding of yak adaptability but also provide a scientific basis for improving disease resistance. By identifying and analyzing immune-related molecular markers, we can uncover candidate genes linked to key immune traits, thereby promoting the accuracy and effectiveness of disease-resistant breeding in yaks at the molecular level.

It is important to note that only female yaks were included in this study. This decision was made during the study design phase for two main scientific reasons. First, immune-related traits are influenced by sex hormones and sex-specific physiological differences. To reduce confounding variability and ensure phenotypic consistency, particularly in the context of complex immune traits, we used a single-sex population. Second, in yak production systems, females are central to herd productivity, maternal immunity, and neonatal health. Improving the genetic basis of immune traits in females can directly contribute to disease resistance and reproductive success. Furthermore, in traditional yak husbandry, herders tend to maintain a higher proportion of females for milk production and limit the number of males to avoid mating-related aggression, which result in a naturally female-biased population structure.

In this study, we observed a predominantly positive correlation between immune indicator phenotypes, suggesting that these immunophenotypes may share common regulatory mechanisms in specific physiological or pathological states. This research represents the first application of genome-wide association study (GWAS) analysis to immune markers in yaks. By conducting a genome-wide association analysis on 192 yaks using SNPs detected through whole-genome sequencing (WGS), we identified several significantly associated SNPs and candidate genes that may be linked to immune traits. Through the annotation of candidate gene regions, we discovered a nonsynonymous mutation associated with Immunoglobulin A (IgA) in the humoral immune response, located within the evolutionarily conserved transcription factor Castor zinc finger 1 (*CASZ1*) gene. *CASZ1*, also known as zinc finger protein 693 (*ZNF693*), plays a crucial role in various embryonic development and physiological processes and has been implicated in several diseases [36,37]. For instance, *CASZ1* regulates the expression of genes associated with cell growth and development while inhibiting cell migration and tumorigenicity in neuroblastoma cell lines [38]. Furthermore, *CASZ1* has been identified as a tumor suppressor in hepatocellular carcinoma [39], and it has been proposed that *CASZ1* may serve as a potential driver or biomarker for certain skin diseases [40]. These findings provide valuable insights into how *CASZ1* influences variations in immune characteristics in yaks.

Among the inflammatory indicators, we identified genes such as lysine methyltransferase 2C (*KMT2C*) and ribosomal protein S6 kinase B1 (*RPS6KB1*). *KMT2C* is a member of the lysine methyltransferase family, and mutations in this gene are associated with the progression, metastasis, and drug resistance of various cancer types. It may play a role in regulating tumor prognosis, immune cell infiltration, and the immune microenvironment. However, the specific molecular mechanisms remain incompletely understood. Existing studies suggest that *KMT2C* may have a potential tumor suppressor effect [41,42]. Notably, *KMT2C* knockout experiments have resulted in social deficits and intellectual disabilities [43]. Additionally, missense heterozygous mutations in *KMT2C* have been reported in children with intellectual disabilities, autism, and mild facial dysmorphism [44]. Further animal experiments support the potential role of *KMT2C* in regulating inflammation. Studies using mouse models have shown that *KMT2C* and *PIK3R3* are significantly upregulated in the epidermis, accompanied by epidermal hyperplasia and inflammatory cell infiltration. However, when a lysine methyltransferase inhibitor (*siKmt2c*) was applied, the symptoms of inflammation and hyperplasia were alleviated, indicating that the inhibition of *KMT2C* can disrupt the immune cycle in immune diseases such as psoriasis [45]. These findings provide a reference for understanding the regulatory role of *KMT2C* in the inflammatory response of yaks and further support the potential application of *KMT2C* in regulating immune-related traits and anti-inflammation. Ribosomal Protein S6 Kinase B1 (*RPS6KB1*) belongs to the family of protein kinases [46], and studies have shown that the mumps virus (MuV) displays faster proliferation in cells lacking *RPS6KB1*, suggesting an inhibitory effect of *RPS6KB1* on viral growth [47]. In addition, *RPS6KB1* has been viewed as a potential biomarker for aggressive malignant lymphoma [48], especially in patients with HIV [49], and has also been associated with the development of multiple sclerosis in Iranian populations [50]. These findings demonstrate a potential regulatory function of *RPS6KB1* in antiviral and immune-related diseases. Our study found that *RPS6KB1* was significantly correlated with the serum level of Haptoglobin (HP) in yaks, suggesting that *RPS6KB1* may have underexplored functions in inflammatory response and immune regulation, which provides new ideas for further understanding the regulatory mechanism of HP.

Cytokines can be classified into several categories, including tumor necrosis factor (TNF), interleukin (IL), interferon (IFN), and others. Various cells can secrete specific cytokines under different physiological conditions, triggering a range of immune responses [4]. In this study, we found that Thrombospondin Type 1 Domain Containing 4 (*THSD4*) exhibited a significant association with Interleukin-6 (IL-6). *THSD4* belongs to the thrombospondin family, which regulates cell attachment, proliferation, and migration. It is homologous to extracellular calcium-binding proteins and plays a crucial role in wound healing, inflammation, and angiogenesis [51]. Pathogenic variants in *THSD4* have been shown to cause thoracic aortic aneurysm and its secondary complications, such as aortic rupture or dissection (TAAD) [52]. Additionally, *THSD4* has been identified as an important gene in a genome-wide association analysis of lung function [53], further supporting its potential significance in inflammation and immune regulation. In addition, KEGG enrichment analysis indicated that *THSD4* and *RPS6KB1* were significantly enriched in the TGF-β signaling pathway. TGF-β signaling can stimulate a variety of cellular responses and plays a crucial role in embryonic development, wound healing, tissue homeostasis, and immune regulation. The dysregulation of TGF-β signaling is characteristic of numerous human diseases, and targeting this pathway may offer broad therapeutic potential [54,55,56]. These findings provide valuable insights for further investigating the regulatory roles of *THSD4* and *RPS6KB1* in immune and inflammatory responses in yaks, and they also highlight the potential application of TGF-β signaling in anti-inflammatory and immune regulation.

In this study, we identified 64 genes associated with humoral immunity, 15 genes linked to inflammation, and 46 genes related to cytokines in yak serum for the first time. From a breeding perspective, these findings provide an important molecular basis for the genetic improvement of health and disease resistance traits in yaks, laying the groundwork for optimizing breeding strategies and enhancing disease resistance. Nevertheless, we acknowledge that the use of a single-sex population (females), while helping to reduce biological variability, may restrict the ability to comprehensively assess sex-specific genetic variation, particularly in traits where immune responses may differ between sexes. This limitation should be addressed in future studies by incorporating sex-balanced populations. In addition, the generalizability of our results may be constrained by several factors, including a limited sample size, the lack of an independent replication cohort, and the inherent logistical challenges of sampling yaks in high-altitude environments. Therefore, while these findings offer valuable preliminary insights into the genetic architecture of immune traits in yaks, they should be interpreted with caution. Nonetheless, the identification of these molecular markers not only deepens our understanding of immune regulatory mechanisms in yaks, but also provides a valuable resource for future precision breeding of health-related traits via marker-assisted selection (MAS). Further research involving larger and more diverse yak populations is needed to verify and extend these findings. High-coverage sequencing and functional validation of candidate loci and genes will also be essential to ensure the reliability of results and provide a more precise genetic foundation for breeding disease-resistant yaks.

## 5. Conclusions

In summary, we identified 323 significant SNPs associated with 10 immune traits and pinpointed 125 candidate genes through a genome-wide association analysis of yak serum immune indicator concentrations. These findings not only unveil the gene network related to humoral immunity, inflammatory responses, and cytokine production in yaks for the first time, but also offer valuable molecular marker resources for the molecular breeding of yak health and disease resistance traits.

## Figures and Tables

**Table 1:** Descriptive statistics of immune traits.

Trait	Mean	Max	Min	SE	CV (%)
IgA (μg/mL)	1153.06	1669.74	831.48	11.97	14.23
IgG (mg/mL)	2.32	3.68	0.49	0.04	22.35
IgM (μg/mL)	2117.54	2930.52	1445.36	18.75	12.17
CRP (mg/L)	13.55	18.90	7.30	0.14	14.03
HP (ng/mL)	400.76	550.96	233.94	3.91	13.38
IL2 (pg/mL)	310.08	438.41	159.40	3.81	16.92
IL4 (pg/mL)	67.60	100.10	25.77	1.08	22.06
IL6 (pg/mL)	431.89	628.51	231.68	4.70	14.93
IFN-γ (pg/mL)	1744.09	2348.44	1193.47	15.68	12.32
TNF-α (pg/mL)	81.23	117.10	53.95	0.84	14.19

Note: IgA = Immunoglobulin A, IgG = Immunoglobulin G, IgM = Immunoglobulin M, CRP = C-reactive protein, HP = Haptoglobin, IL2 = Interleukin-2, IL4 = Interleukin-4, IL6 = Interleukin-6, IFN-γ = Interferon γ, and TNF-α = Tumor necrosis factor α.

## Data Availability

The data presented in the study are deposited in the NCBI repository, accession number PRJNA1188071.

## Supplementary Materials

The following supporting information can be downloaded at: https://www.mdpi.com/article/10.3390/ani15142114/s1, Supplementary Figure S1. Number of SNPs within 1 Mb windows. Supplementary Table S1. Raw measurements of 10 immune traits; Supplementary Table S2. Annotation results for all SNPs; Supplementary Table S3. Significant SNPs associated with immune traits; Supplementary Table S4. Results of enrichment by GO analysis for the candidate genes of immune traits; Supplementary Table S5. Results of enrichment by KEGG analysis for the candidate genes of immune traits.
