# Supplementary figures and images for: Genome-Wide Association Study of Immune Indices in Yaks
Source: Animals (Basel). 2025 Jul 17;15(14):2114. doi: 10.3390/ani15142114 (PMC12291813; doi:10.3390/ani15142114)

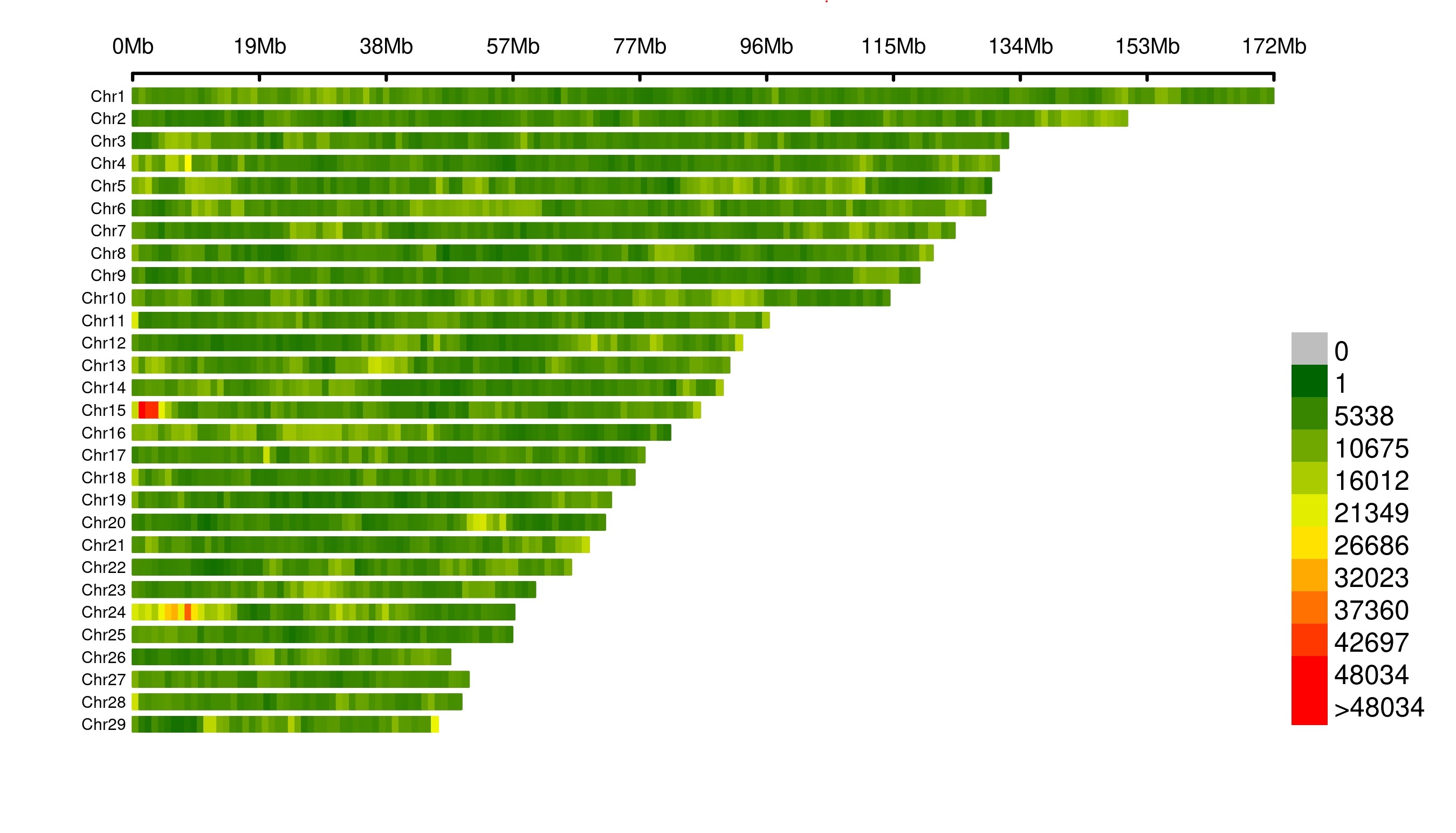

Supplement: Supplementary file 1 [file animals-15-02114-s001.zip › Supplementary Fig. S1.jpg]

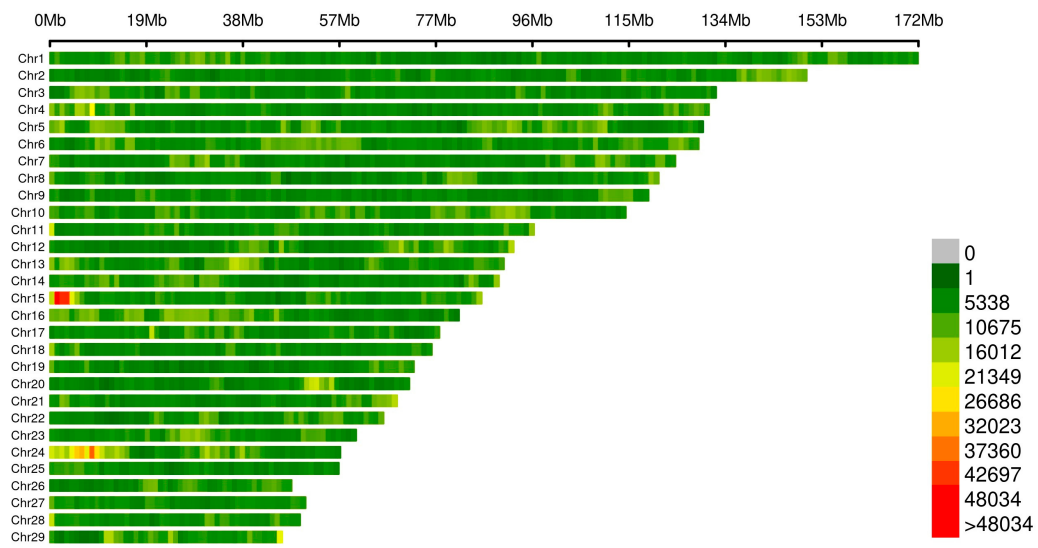

**Supplementary Fig. S1.** Number of SNPs within 1 Mb windows.

Supplement: Supplementary file 1 [file animals-15-02114-s001.zip › Supplementary Fig.pdf]
